# Sudden unilateral corneal clouding in diabetic patient: A case report and literature review

**DOI:** 10.1097/MD.0000000000033919

**Published:** 2023-06-02

**Authors:** Man Xu, Shujuan Wu, Xiaoguang Niu

**Affiliations:** a Aier Eye Hospital of Wuhan University, Wuhan, Hubei Province, China.

**Keywords:** anterior chamber exudation, case report, complications, corneal opacity, diabetic

## Abstract

Corneal opacity can be caused by various disease. Generally, the opacity gradually increases as the disease progresses. Sudden corneal opacity is mainly caused by corneal trauma, toxic drugs entering the cornea, or acute edema of the keratoconus. However, sudden corneal opacity caused by diabetes has not been reported.

**Patient concerns:**

A 60-year-old man reported blurred vision and the black eye became white in appearance in the left eye for 5 days. The patient had a history of diabetes which had not been treated.

**Diagnoses:**

He underwent slit-lamp examination, anterior segment optical coherence tomography, ultrasound bio microscopy, B-mode ultrasound, corneal endothelial examination, random blood glucose testing, and other examinations. The diagnosis of Diabetic Keratopathy was made.

**Interventions:**

Topical glucocorticoids and dilating eye drops were administered and undergo blood sugar control treatment.

**Outcomes:**

The corneal of the patient was completely transparent in a few days, and the flocculent exudation in the anterior chamber disappeared.

**Lessons:**

Although diabetes generally causes chronic corneal edema, acute corneal edema may also occur when blood sugar is poorly controlled. Therefore, when we see sudden corneal opacity without obvious incentives, we must consider systemic diseases, especially diabetes.

## 1. Introduction

Corneal opacity can be caused by various pathological factors, such as mechanical, immune-related, and infectious factors.^[1]^ Corneal opacity with different etiologies also has different morphological characteristics. The inflammatory opacity caused by pathogenic microorganisms such as bacteria, fungi, and viruses is mainly discoid opacity of the cornea. However, corneal opacity gradually occurs and worsens with the development of the disease, and corneal opacity presents a chronic progression process. Sudden corneal opacification is common in trauma or drug toxicity. Mandal once reported a case of sudden corneal clouding in a patient with infantile glaucoma^[2]^. In the acute stage of keratoconus, there is sudden corneal opacity and edema in the central area due to rupture of the Descemet’s membrane. Most corneal opacities may still have varying degrees of opacity remaining in the cornea even after disease control. We recently treated a patient with sudden corneal opacity, who exhibited diabetes with poor blood glucose control and the cornea is completely transparent after treatment. The report is as follows:

## 2. Case report

**Figure 1. (:**
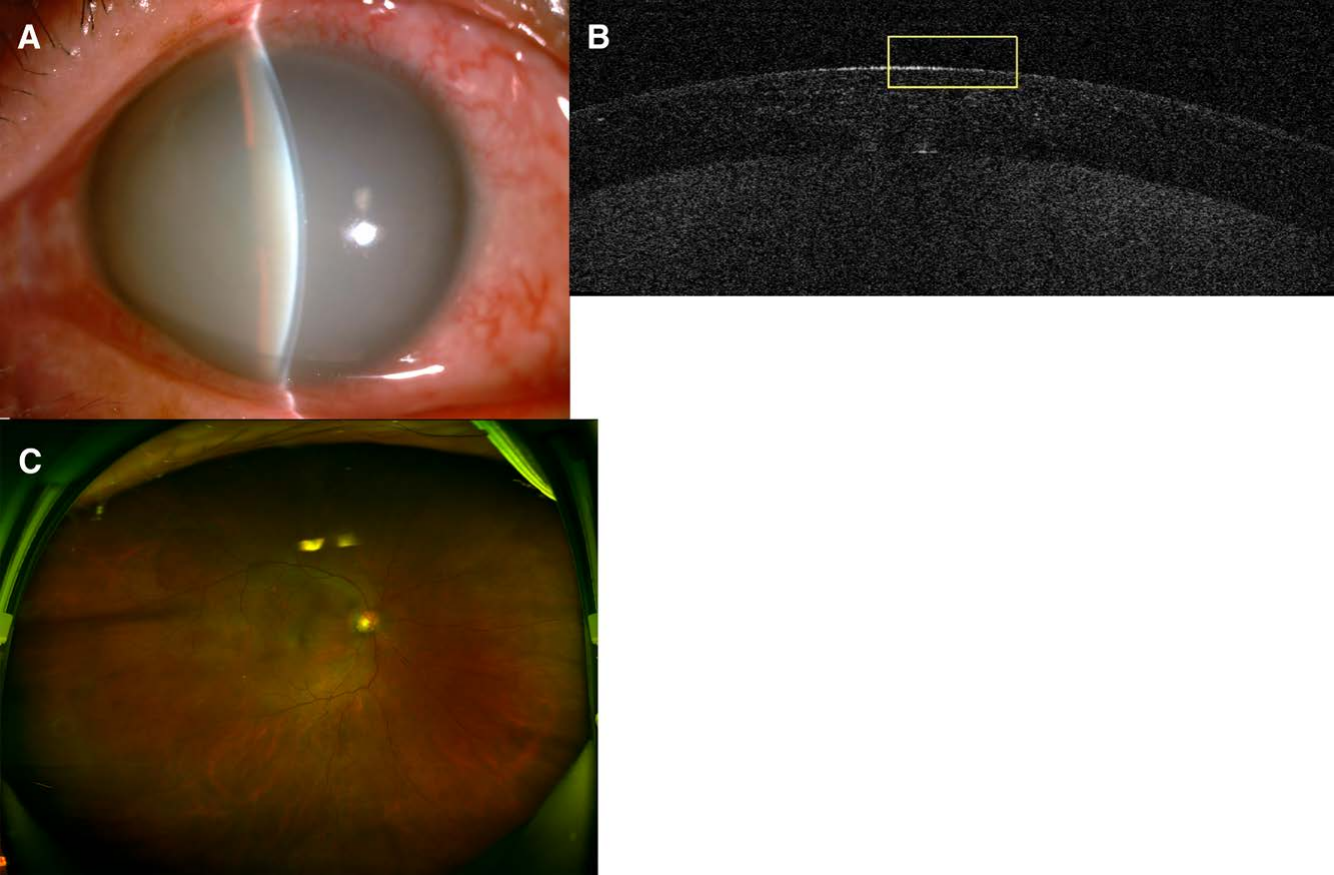
A) Anterior segment photograph of the left eye. Uniform edema and white opacity in the deep stroma in the whole corneal of the left eye. (B) Anterior segment optical coherence tomography (OCT) of the left eye. The corneal was uniformly thickened, and a high-reflection shadow could be seen. (C) Fundus photography of the right eye. Scattered points of bleeding in the retina of the right eye.

**Figure 2. (:**
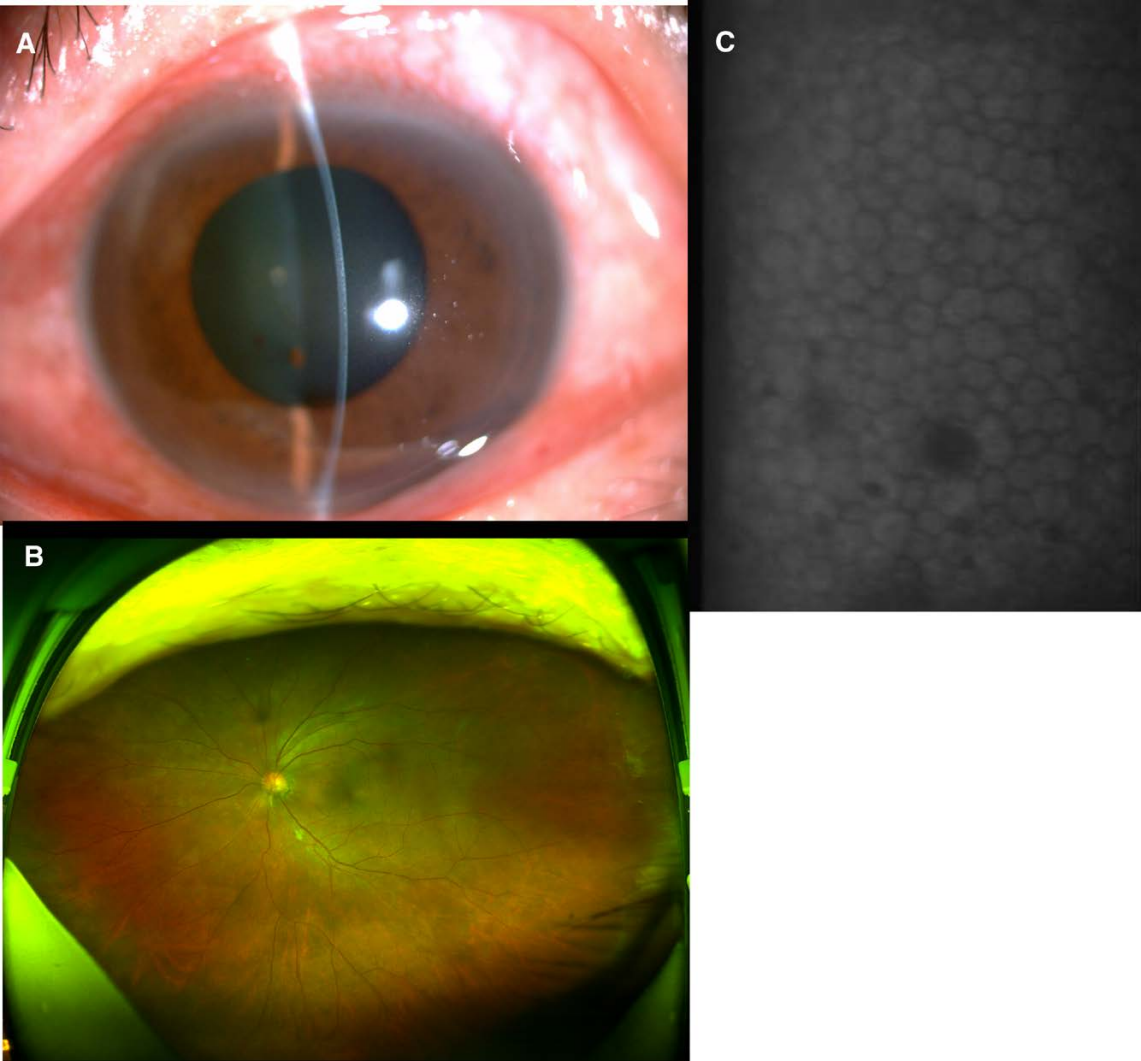
A) Anterior segment photograph of the left eye after treatment. Corneal became transparent, Iris pigmentation on the anterior surface of the lens. (B) Fundus photography of the left eye after treatment. Scattered points of bleeding in the retina of the left eye. (C) The endothelium of the corneal. The corneal endothelium of both eye was varying in size.

A 60-year-old men, came to our hospital on March 10, 2022. The patient reported blurred vision and the black eye became white in appearance in the left eye for 5 days. The patient has no previous history of eye disease, and had a history of Type 2 diabetes with no systematic treatment. Visual acuity: 20/40 OD and 20/200 OS; intraocular pressure: 16.0 mm Hg OD and 17.0 mm Hg OS; uniform edema in the whole corneal of the left eye; white opacity in the deep stroma (Fig.1A); a posterior pole examination was limited by corneal opacity. The right eye had no corneal edema. Anterior segment optical coherence tomography (Fig.1B) showed that the corneal of the left eye was uniformly thickened, and a high-reflection shadow could be seen. Ultrasound bio microscopy in the left eye showed a small amount of flocculent exudation on the anterior surface of the lens. Fundus photography showed scattered points of bleeding in the retina of the right eye (Fig.1C). The patient’s random blood glucose 26 mmol/L. Atropine eye gel, tobramycin dexamethasone eye drops and ointment were administered to the patient. Additionally, the patient was asked to control his blood glucose and return on 3 days. The patient came to the hospital for a follow-up visit on March 19, 2022. The main result was that the vision of the left eye had improved, and the blurred vision had cleared after 3 days of medication. Ophthalmic examination revealed the following: visual acuity: 20/40 OS; the corneal edema of the left eye had resolved and turned transparent completely; flocculent exudate disappeared (Fig. 2A); and scattered points of bleeding on the retina (Fig. 2B). The endothelium of the corneal was varying in size (Fig. 2C). Random blood glucose is 13 mmol/L. Atropine ophthalmic gel was stopped, and the patient has not been reexamined since then. A month later, the patient was followed up by phone and reported no ocular discomfort.

## 3. Discussion

While we cannot prove that these symptoms were caused by or directly related to high blood glucose, we did not find any other plausible cause that could explain these ophthalmic signs. The patient had poorly controlled hyperglycemia; therefore, we considered that the sudden corneal opacity was related to the hyperglycemia. Diabetes can cause pathological changes in various tissues of the eye. Corneal diseases caused by diabetes are also quite common, occurring in up to 70% of diabetic patients. Diabetes can affect all layers of corneal tissue. The most important effects are corneal epithelial abnormalities, such as superficial punctate keratitis, delayed wound healing, recurrent corneal erosion, ulcers with difficulty healing, and corneal edema caused by decreased corneal epithelial barrier function. The corneal sensitivity of diabetic patients is generally decreased, and the degree of decreased sensitivity is related to the severity of the disease.^[3]^ In vivo confocal microscopy has shown that the corneal nerve fiber curvature in diabetic patients is increased and that the density, length and branch density are abnormal.

The corneal thickness is increased in patients with diabetes compared with that in nondiabetic patients, but this increase in corneal thickness is chronic. On the one hand, the cross-linking of corneal stromal collagen caused by glycosylation increases the hardness and thickness of the cornea; on the other hand, the increases thickness is related to the swelling pressure of the corneal stroma and dysfunction of endothelial cell pumps.^[4]^ At present, there have been no reports of acute corneal edema.

The tear film glucose content is 4 times higher in patients with diabetes than in nondiabetic patients, and the glucose content in the posterior 2/3 of the corneal stroma is higher than that in the anterior 1/3 of the corneal stroma. The water absorption capacity of the deep stroma of the cornea is stronger than that of the shallow stroma,^[5]^ which may explain why the white haze we observed is mainly located in the deep stroma of the cornea.

Recent studies have found not only that the number of corneal endothelial cells in diabetic patients is lower than that in nondiabetic patients but also that cell pleomorphism and variability are increased.^[6]^ After cataract phacoemulsification, the density of corneal endothelial cells in diabetic patients is significantly lower than that in nondiabetic patients, and the endothelial permeability is significantly increased. Additionally, the increase in the central corneal thickness has been found to be significantly higher and the postoperative recovery slower in diabetic patients than in nondiabetic patients, mainly due to the decline in endothelial function reserve and stress response ability caused by diabetes.^[7]^ In our case, the patient had normal corneal endothelial cell density in both eyes, but varying in size. Therefore, we believe that the patient may had poor corneal endothelial function.

Anterior chamber exudation indicating the presence of anterior uveitis. Many studies have shown that diabetes is related to idiopathic anterior uveitis^[8]^; while the mechanism has not been fully elucidated, it may be related to autoimmune factors and destruction of the blood-eye barrier or eye ischemia caused by diabetes.^[9]^ Recently, some scholars have proposed that it is related to the cross reaction of serum insulin antibodies in patients with diabetes.^[10]^

In terms of treatment, patient was given local ophthalmic glucocorticoid drugs. After treatment, the transparency of the cornea recovered, and the vision improved, which may be related to endothelial cell pump function activation induced by the glucocorticoids.^[11]^ Due to the anti-inflammatory effect of glucocorticoids, the flocculent exudation in the patient’s anterior chamber had completely disappeared by the follow-up visit.

It is worth noting that although the patient had severe acute corneal edema, the retinopathy was not serious. There were only a few scattered points of bleeding on the retina of both eyes, and no hard exudates or serious proliferative changes were found. Therefore, keratopathy in patients with diabetes may occur earlier than or at the same time as retinopathy, and the two are not synchronized.

## 4. Conclusion

The mechanism of the influence of diabetes on the cornea has not been fully clarified, and acute corneal edema caused by diabetes is not common. It is very important to determine the cause of corneal edema according to its morphology. Such as this patient, As a result of not paying attention to the control of blood glucose, acute corneal edema occurred. If the poor glycemic control continued, whether repeated acute corneal edema under hyperglycemia has a great impact on the corneal endothelium and corneal stroma or whether there will be other complications remains to be observed. Various eye changes are caused by diabetes. For patients with diabetes, it is necessary to strictly control blood glucose and conduct regular examinations to minimize the impact of eye-related and systemic complications.

## Author contributions

**Writing – original draft:** Man Xu.

**Writing – review & editing:** Shujuan Wu, Xiaoguang Niu.
